# Brain banks in Latin America: Infrastructure for diagnosis, research, and scientific equity in Mexico and the Caribbean

**DOI:** 10.1002/alz.70819

**Published:** 2025-11-09

**Authors:** Nabil Itzi Luna‐Viramontes, Alejandra Morlett‐Paredes, Itzcoatl Ordoñez‐Lozano, Fidel de la Cruz‐López, Vanessa Esmeralda Gonzalez‐Chavez, Genaro Vargas‐Hernández, E. G. Pérez‐Pérez, Rogelio E. Méndez‐Llaca, Ignacio Villanueva‐Fierro, Roció Ortiz‐Butron, Mario Hernandes‐Alejandro, Linda Garcés‐Ramírez, José Luna‐Muñoz

**Affiliations:** ^1^ National Dementia BioBank, AMPAEYDEN A.C. Estado de México México; ^2^ Departamento de Fisiología. Escuela Nacional de Ciencias Biológicas Instituto Politécnico Nacional Ciudad de México México; ^3^ Deparment of neurociences University of California La Jolla California USA; ^4^ Programa Educativo en Ciencias y Tecnologías Avanzadas Universidad Politécnica de Pachuca Zempoala Hidalgo México; ^5^ Programa Educativo Posgrado en Biotecnología Universidad Politécnica de Pachuca unidad Zempoala Pachuca México; ^6^ Dirección de Investigación Innovación y posgrado. Universidad Politécnica de Pachuca Zempoala Hidalgo México; ^7^ Instituto Politécnico Nacional. CIIDIR. Unidad Durango. Becario COFAA Durango México; ^8^ Departamento de Bioingeniería Unidad Profesional Interdisciplinaria de Biotecnología del Instituto Politécnico Nacional México México; ^9^ Banco Nacional de Cerebros‐UNPHU, Universidad Nacional Pedro Henríquez Ureña Santo Domingo República Dominicana

**Keywords:** Alzheimer's disease, amyloid beta, brain bank, dementia, neuropathology, tau protein

## Abstract

The establishment of brain banks is a strategic initiative to advance research on neurodegenerative diseases in Latin America. This article highlights the development, operations, and multidisciplinary framework of the National Dementia BioBank in Mexico and the National Brain Bank in the Dominican Republic. Both centers focus on the molecular characterization of pathological proteins central to Alzheimer's disease, such as tau and amyloid beta. Standardized protocols have been implemented for tissue donation, preservation, and *post mortem* analysis. Beyond supporting diagnostics and research, these centers promote scientific literacy and public engagement through traveling exhibitions and outreach initiatives. Together, these brain banking efforts enhance regional scientific capacity, strengthen international collaboration, and advance the representation of historically underserved populations. Their work contributes to a more equitable and globally relevant neuroscience landscape, positioning Latin America as a growing contributor to translational research in aging and dementia.

**Highlights:**

- Creation of two national brain banks in Mexico (National Dementia BioBank) and the Dominican Republic (National Brain Bank–UNPHU) with standardized protocols for donation, preservation, and *post mortem* analysis of tau and amyloid beta under international parameters.
- Integration of advanced histopathological and molecular diagnostic platforms combining classical stains (H&E, Bielschowsky) with immunoperoxidase, multiplex immunofluorescence staining, confocal microscopy, and AI‐powered automated analysis for quantification and recognition of topographic patterns.
- Design of an inclusive tissue collection model that prioritizes the genomic and sociocultural representation of mestizo, Afro‐Caribbean, and Indigenous populations, reducing Eurocentric bias and enhancing the global validity of studies on Alzheimer's and other dementias.
- Development of a culturally sensitive, ethical, and neuroethical framework, with an informed consent process accessible in multiple languages and literacy levels, and safeguarding confidentiality through rigorous data coding.
- Implementation of innovative outreach strategies (traveling exhibitions, augmented/virtual reality, public lectures) to promote a culture of brain donation and democratize knowledge about neurodegeneration in diverse communities.

## 1 INTRODUCTION

The establishment of brain banks has become a cornerstone of scientific infrastructure in countries striving to lead biomedical and clinical research on the central nervous system.^1^ In Latin America, a rapid demographic shift toward an aging population has elevated the urgency of addressing neurodegenerative diseases such as Alzheimer's disease and related dementias.^2^ These conditions pose an urgent public health concern. Addressing them requires sustained scientific investment. Importantly, dementia risk factors vary across populations, underscoring the need for region‐specific data to inform effective public health policies aimed at reducing the incidence of major cognitive disorders.^3^

Despite this urgency, access to *post mortem* human brain tissue remains limited in many Latin American nations. This scarcity presents a critical obstacle to the advancement of translational neuroscience, hindering efforts to characterize population‐specific molecular features and limiting opportunities for diagnostic, therapeutic, and educational research. In response, the development of regional brain banks is essential not only to support clinical and research infrastructure but also to foster community engagement and participation in biomedical science.

Mexico and the Caribbean represent a strategic region for such initiatives. Beyond their rising burden of neurodegenerative diseases, these areas offer rich genomic diversity,^2^ growing academic capacity, and a timely opportunity to strengthen scientific autonomy in contrast to historically centralized research models.^4^ The creation of specialized neurobanks in these regions facilitates the preservation of high‐quality brain tissue under internationally accepted standards, promotes national and international research collaborations, contributes to workforce development, and supports public engagement through culturally tailored education campaigns on brain and biospecimen donation.^5^, ^6^ Together, these efforts represent a model for integrating science, bioethics, and social equity in the pursuit of translational neuroscience in Latin America.

### 1.1 Neurodiversity and underrepresentation in global neuroscience: the transformative role of brain banks in Latin America

Contemporary neuroscience has made significant advances in understanding the cellular, molecular, and genetic mechanisms underlying neurodegenerative and psychiatric disorders. However, much less attention has been paid to neurodevelopmental alterations.^7^ Moreover, a considerable portion of these advances is based on studies conducted in relatively homogeneous populations, primarily of European or North American origin.^8^ This limited diversity introduces a bias in global neuroscience, where major population groups remain underrepresented, ultimately affecting diagnostic accuracy and the development of culturally appropriate clinical interventions.^9^

**FIGURE 1:**
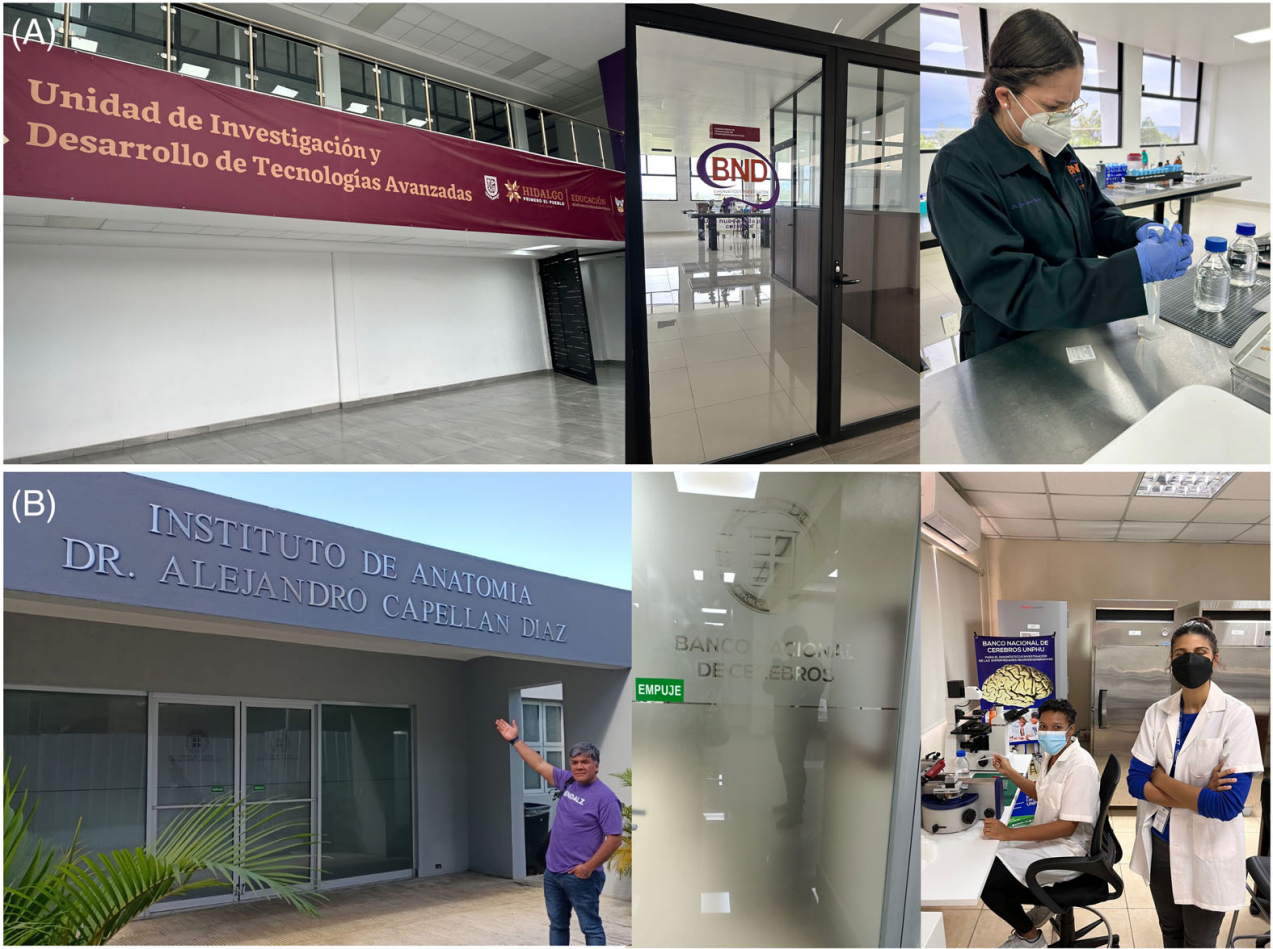
Infrastructure for research on neurodegenerative diseases. (A) The National Dementia BioBank is housed within the Research and Development Unit for Advanced Technologies at the Universidad Politecnica of Pachuca in Zempoala, Hidalgo, Mexico. This facility processes donated tissue for research purposes, reflecting both scientific and diagnostic efforts. (B) The National Brain Bank is located within the Department of Anatomy at the Universidad Nacional Pedro Henríquez Ureña in Santo Domingo, Dominican Republic, where specialized personnel conduct research activities focused on dementias.

Regional brain banks play a critical and strategic role not only as hubs for research and diagnosis but also as instruments of inclusion. In regions such as Mexico and the Caribbean^8^, ^10^ (Figure 1), populations are characterized by rich genetic, cultural, and linguistic diversity.^11^ The establishment of brain banks in these areas will enable the systematic collection of brain tissue, organ fragments, and fluids from mestizo, Afro‐descendant, and Indigenous communities. Recent studies led by researchers from outside the region are beginning to recognize the importance of conducting research within these populations.^12^ This inclusion is not only ethically and socially necessary but also scientifically essential. Research has identified genetic variants linked to Alzheimer's disease (AD) and other neurological conditions in Latin American populations,^13^, ^14^ suggesting that epigenetic mechanisms may lead to population‐specific disease expression.^15^ Such findings underscore the need for access to biological samples from these communities, something that can only be achieved through the development of local brain banks. These institutions are uniquely positioned to obtain informed consent in culturally sensitive ways while preserving the sociolinguistic integrity of each case.^5^, ^6^

Importantly, the brain banks in Latin America also emphasize the documentation of broader social determinants, such as chronic poverty, discrimination, intergenerational stress, and unequal access to education, which may influence neurobiological pathways, including metabolic disruption and aberrant synaptic pruning. Studying these processes in historically marginalized populations is crucial for a deeper understanding of the pathophysiology of the nervous system within its social and environmental context.^16^

The role and importance of these brain banks extend far beyond the passive preservation of samples. We envision them as active research platforms designed to foster the development of population‐specific bioprofiles.^17^ Over time, this work will support the creation of regional genomic databases, much like the efforts led by the brain bank in Colombia.^18^, ^19^ There, researchers have built a dedicated infrastructure for identifying cases of familial AD and have discovered mutations that delay symptom onset into later life stages in certain genetic profiles.^19^, ^20^ Such efforts support the decolonization of biomedical knowledge and promote the development of a more globalized neuroscience, one that seeks to embrace the complexity and diversity of the human brain. The impact of these initiatives is particularly significant when they include Afro‐Caribbean communities and Indigenous populations. Brain banks are transforming the relationship between science and society. What was once an exclusive and inaccessible process is now becoming a space for empowerment and collective memory. In this context, *post mortem* donation is increasingly viewed as an act of legacy, dignity, and contribution to the future.^21^

### 1.2 Neuroethics and *post mortem* tissue donation management: foundations for a conscious science in Latin America

The donation of *post mortem* tissues, such as brain and bodily fluids, for research purposes constitutes a profound act of generosity by donors and/or their families.^21^, ^22^ Such contributions provide invaluable opportunities to advance the field of neuroscience. However, these practices must be carefully guided by ethical, cultural, and legal considerations, particularly within the Latin American context, which is marked by structural inequality, sociocultural diversity, and institutional challenges.^23^ In this setting, it is essential to critically examine the moral implications of knowledge production and the use of technologies applied to the human brain through the framework of neuroethics.^24^, ^25^ This ethical lens is necessary for the proper regulation and governance of donations within brain banking initiatives. Informed consent, in particular, must be upheld as a fundamental ethical expression of the individual's right to autonomy and bodily integrity even after death.^26^, ^27^

### 1.3 Informed consent

Informed consent for the donation of brain and other tissues must not be reduced to a purely legal formality; rather, it should be understood as an ongoing, transparent, and culturally sensitive dialogue.^28^ In Latin American countries, it is essential to design and implement consent materials that are appropriate for varying levels of literacy, linguistic diversity, and cultural worldviews.^29^ Families should receive clear, accessible information regarding the purpose of tissue donation, its scientific and societal value, and the eventual use of biological samples. Additionally, mechanisms for advance consent, obtained while the individual is still cognitively able to decide, should be established.^30^ In cases where the patient has already experienced cognitive decline, substitute or proxy consent must be considered. In all cases, the principle of autonomy must be upheld as the foundation of ethical practice.^31^

### 1.4 Confidentiality and data protection

Protecting the confidentiality of donor identities is essential to safeguarding personal privacy and preventing the misuse of biological materials.^32^ Brain banks must implement rigorous protocols for the coding and de‐identification of specimens and medical records. Access to identifying information must be strictly controlled, with encrypted storage systems and limited personnel access to ensure compliance with data protection standards. These practices are vital to maintaining public trust and upholding ethical standards in the management of *post mortem* tissue donations.^32^, ^33^

### 1.5 Equity, non‐maleficence, and justice in research

In the context of longstanding structural inequalities, it is imperative to ensure that brain banking initiatives are guided by the principles of equity, non‐maleficence, and justice.^34^ These principles demand proactive efforts to avoid perpetuating historical disparities in access to research resources and scientific benefits. At the National Dementia BioBank (BND, for its initials in Spanish) in Mexico and the National Brain Bank at UNPHU (BNC‐UNPHU, for its initials in Spanish) in the Dominican Republic, special emphasis is placed on equitable access to donated tissue for both national and international researchers. Priority is given to research projects that are likely to yield tangible benefits for donor populations.

To ensure ethical reciprocity, findings derived from the use of donated brain tissue should be disseminated back to the communities of origin through tailored outreach, education, and public health programs.^35^, ^36^, ^37^ Neuroethics and the governance of *post mortem* tissue must serve as the ethical foundation of every brain bank that seeks to advance justice, legitimacy, and social impact.^33^, ^38^ In Latin American countries, where science often unfolds within contexts marked by historical injustice and social vulnerability, neuroethics becomes a necessary bridge between cutting‐edge research and the respect for human dignity.

### 1.6 Public education and scientific culture surrounding brain donation: democratizing knowledge, honoring legacy

**FIGURE 2:**
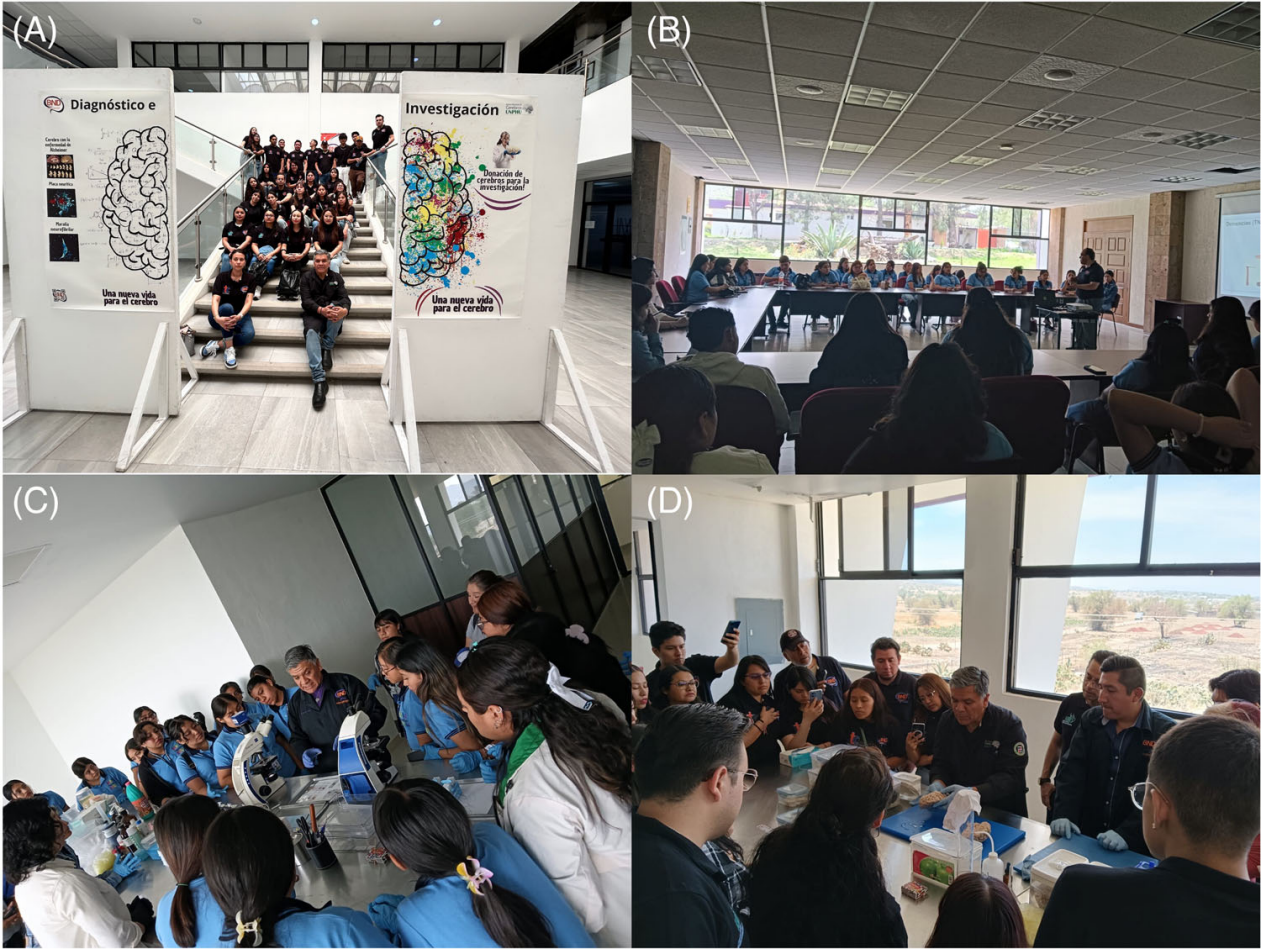
Educational event focused on public outreach and active learning about neurodegenerative diseases. (A) Guided tour of brain bank, a space dedicated to research and diagnosis. (B) Students attend an introductory lecture on the scientific and social projects developed in the laboratory. (C) Observation of histopathological samples through light microscopy, highlighting alterations associated with neurodegenerative diseases. (D) Applied neuroanatomy class discussing structural brain changes linked to various neurodegenerative diseases.

One of the key challenges encountered in the development of these brain banks has been limited financial resources; however, an equally significant barrier has been the prevailing cultural attitudes toward brain donation for research.^38^, ^39^ Cultural resistance is often rooted in myths, fears, and a lack of awareness about *post mortem* brain donation, even among healthcare professionals.^23^, ^40^, ^41^ This resistance is further compounded by misinformation and a disconnect between science, society, and collective memory.^38^ In response, we have implemented strategies to foster a critical, empathetic, and participatory scientific culture surrounding brain donation for research. These efforts aim to bridge the gap between scientific knowledge and public understanding, promoting trust, transparency, and long‐term engagement with the communities we serve (Figure 2).

### 1.7 Public education as a policy of dignification

Educational campaigns on brain donation not only should emphasize the scientific value of tissue for laboratory‐based research. They must also address the symbolic dimension of donation. Donating is an act of legacy, generosity, and a commitment to building a better future.^27^, ^36^, ^42^ We have worked to humanize brain donation for research by highlighting the stories of donors and their families and by incorporating the voices of researchers who explain in clear, accessible, and non‐stigmatizing language how donated tissue contributes to knowledge that benefits everyone. These efforts acknowledge the emotional weight of death and grief, honoring both patients and their loved ones in the process.

### 1.8 Traveling exhibitions and public engagement: from laboratory to society

**FIGURE 3:**
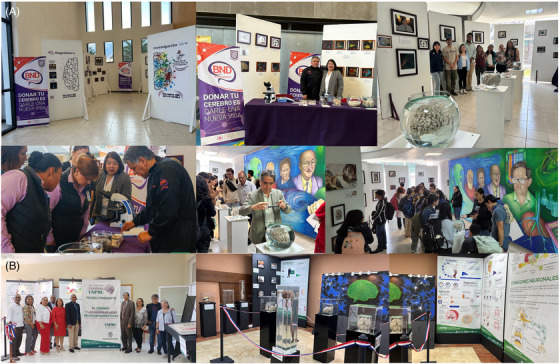
Traveling exhibitions. (A) Traveling exhibitionsin Mexico. (B) Traveling exhibition in Dominican Republic. The exhibition integrates science, culture, and education to raise awareness about brain donation and neurodegenerative diseases, bringing knowledge closer to the public in an inclusive and interactive manner.

The traveling exhibition has played a pivotal role in the outreach efforts of both the BND in Mexico and the BNC‐UNPHU in the Dominican Republic. These exhibitions have been presented at scientific conferences, universities, and public events such as Brain Awareness Week, World Brain Day, and World Alzheimer's Day. As part of these efforts, real human brains preserved in fixatives have been displayed, alongside infographics explaining brain anatomy, neurodegenerative diseases, risk factors for dementia, and strategies for preventing sporadic AD. Photographs of anatomical changes and hallmark lesions in various neurodegenerative disorders are used to illustrate why studying the human brain is essential for understanding these conditions. Interactive augmented reality components have also been developed, creating immersive educational spaces (Figure 3).

These initiatives are not merely decorative or promotional; they are acts of justice that promote equity and ethical knowledge dissemination. In addition, university groups regularly request guided tours of the BND and BNC‐UNPHU. These visits include lectures and presentations on current research efforts, emphasizing the return of scientific knowledge to the very communities that make it possible through tissue donation (Figure 2). Fostering a culture of brain and tissue donation for research in Latin America requires more than infrastructure and regulatory frameworks; it calls for a profound cultural transformation. Donated tissue must not be seen as medical waste but rather as a valuable archive that helps heal others and deepens our understanding of the human condition.

### 1.9 Public education and scientific literacy are the keys to making this vision a lasting reality

#### 1.9.1 Technological innovation at BND and BNC‐UNPHU

The development of the BND in Mexico and the BNC‐UNPHU in the Dominican Republic has embraced cutting‐edge technologies across multiple stages, from tissue preservation to data acquisition for both diagnostic and research purposes. This integration of innovation ensures scientific relevance, ethical traceability, and operational sustainability.

### 1.10 Brightfield microscopy for diagnostic purposes

**FIGURE 4:**
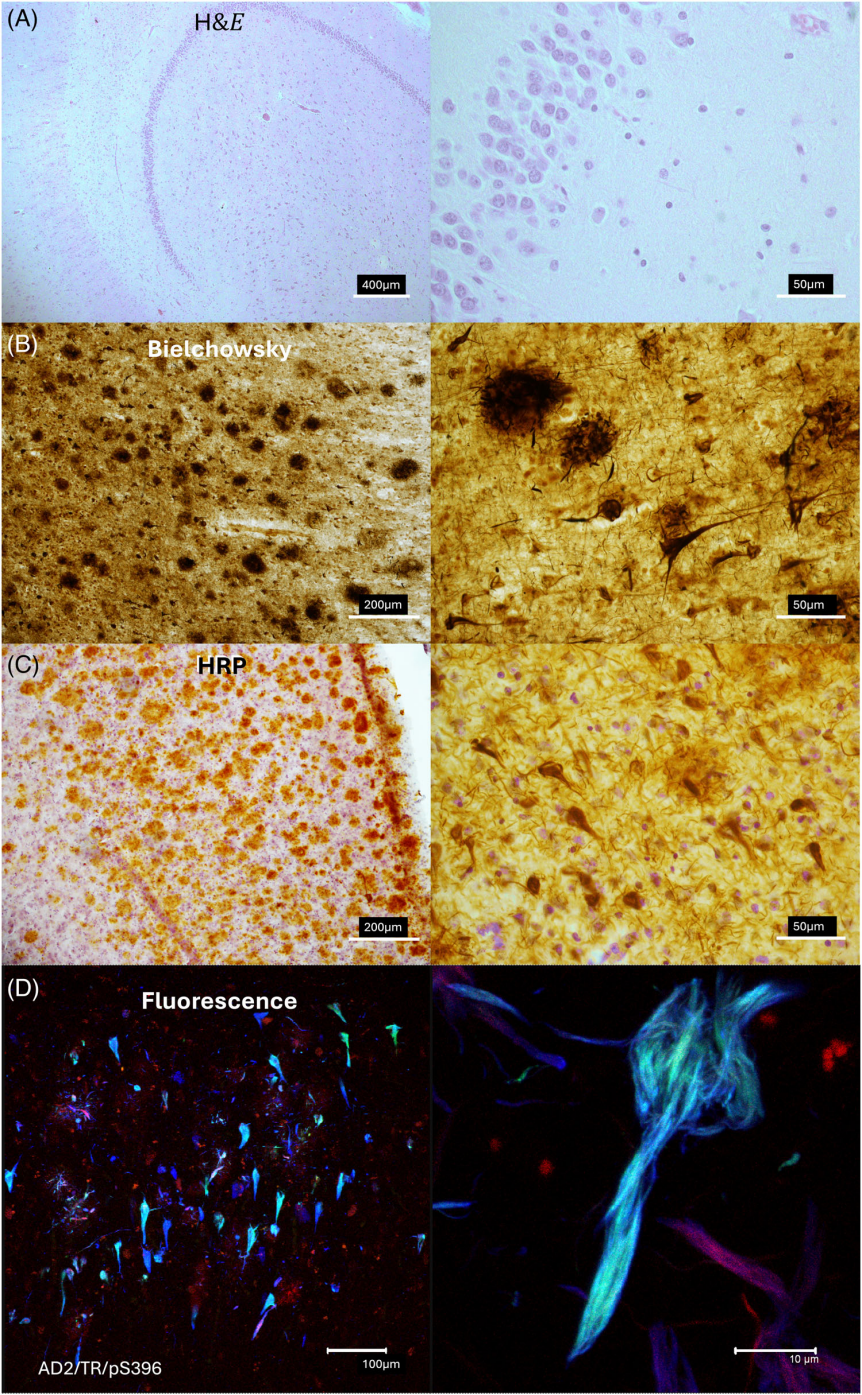
Comparative representation of histopathological techniques used in the study of Alzheimer's disease and other dementias. (A) General hematoxylin and eosin (H&E) staining to assess tissue cytoarchitecture. (B) Bielschowsky silver staining to highlight amyloid plaques and neurofibrillary tangles. (C) Immunoperoxidase technique for detecting pathological proteins such as amyloid beta and tau in Alzheimer's disease. (D) Fluorescence microscopy and confocal analysis to visualize intramolecular relationships among different tau species, including phosphorylated, truncated, and fibrillar forms.

Brightfield microscopy is a widely employed technique in diagnostic neuropathology. It involves the visualization of stained tissue sections using transmitted light, allowing high‐resolution morphological examination. These stained slides can be digitized to create archival records, which also support diagnostic interpretation and the development of artificial intelligence (AI) applications.^43^ In our workflow, we perform hematoxylin and eosin (H&E) staining (Table 1) to assess general cellular architecture, including atrophic regions, neuropil vacuolization, and gliosis. While this stain does not clearly reveal neurofibrillary tangles or neuritic plaques, it provides an overview of the tissue's condition and guides the targeted sampling of specific lesions (Figure 4A).

**TABLE 1:** Activities and techniques used for the studies conducted on specimens obtained through donation to the diagnóstic and reseach units BND and BND‐unphu.

Characteristics	Brightfield microscopy	Immunofluorescence
Image type	2D, transmitted light	3D, optical sectioning
Stain permanence	High – permanent stains	Low – temporary fluorescent staining
Staining method	Chromogenic (e.g., DAB, silver, H&E)	Fluorescent dyes (fluorochromes at different wavelengths)
Clinical diagnostic use	Standard (e.g., NIA‐AA criteria)	Complementary/advanced research
Automated quantitative analysis	Compatible after digital scanning	Compatible with reconstruction software

Abbreviations: DAB, diaminobenzidine; H&E, hematoxylin and eosin; NIA‐AA, National Institute on Aging‐Alzheimer's Association.

### 1.11 Bielschowsky silver stain

This silver staining technique is used to visualize hallmark pathological features of AD, including neurofibrillary tangles, neuritic plaques, and axonal degeneration.^44^, ^45^ Silver salts bind to fibrillar aggregates, revealing lesions within neuronal somas and extracellular deposits. This approach enables the identification and distribution mapping of pathological aggregates in sampled areas. However, it does not determine the specific protein composition of these lesions (Figure 4B).

### 1.12 Immunohistochemical staining

This technique utilizes antibodies directed against proteins or peptides associated with neurofibrillary tangles (such as tau protein) and amyloid plaques (amyloid beta [Aβ]). Additional markers such as glial fibrillary acidic protein (GFAP) and IBA1 are used to identify astroglial and microglial cells, respectively. These immunostains are developed as permanent preparations, stored at room temperature, and analyzed using specialized software that allows for digital quantification of lesions.^46^ This enables the mapping of pathological progression according to Braak and Braak staging criteria^47^, ^48^ (Figure 4C).

### 1.13 Multiplex fluorescent staining and confocal microscopy

Multiplex fluorescent staining involves antibodies that target proteins linked to neuropathological lesions, combined with fluorescent dyes. Confocal microscopy is then used to detect multiple markers at different wavelengths, allowing the visualization of molecular interactions such as tau aggregation and polymerization, as well as Aβ deposition (Table 1).^49^, ^50^, ^51^, ^52^ This method provides high‐resolution, three‐dimensional (3D) imaging of sections up to 100 µm thick, making it particularly well suited for studying molecular relationships and validating biomarkers in neuroscience research (Figure 4D).

### 1.14 AI and automated neuropathological analysis

The integration of AI into the analysis of histopathological sections stained using immunoperoxidase (Figure 4C) and immunofluorescence (Figure 4C) techniques marks a significant shift from qualitative to quantitative assessment in brain banks. At both the BND in Mexico and the BNC‐UNPHU in the Dominican Republic, AI‐based automated analysis systems are being implemented to enhance diagnostic efficiency and democratize access to cutting‐edge technologies, which remain underrepresented in the biomedical innovation landscape of the Caribbean.

These brain banks employ a range of staining methods, including fluorescent, peroxidase‐based, and silver staining, supported by AI‐powered tools for automatic segmentation of neuropathological hallmarks (e.g., amyloid plaques, neurofibrillary tangles, Lewy bodies, Pick bodies). Morphometric quantification is performed across distinct brain regions (e.g., lesion density per square millimeter, staining intensity), along with pattern recognition aligned with diagnostic scales such as Consortium to Establish a Registry for Alzheimer's Disease and Braak staging. The design of regionally trained AI models is a critical innovation, particularly given the unique admixture, African ancestry, and environmental exposures in these populations. This approach has the potential to reveal distinct clinico‐pathological subtypes of neurodegenerative diseases that are currently understudied.

### 1.15 Omics applications in *post mortem* brain research

Both brain banks are advancing toward the integration of high‐throughput omics technologies to enhance the molecular characterization of neurodegenerative diseases. Among these, spatial transcriptomics will allow for the precise mapping of gene expression patterns within anatomically defined brain regions, yielding insights into the spatial organization of transcriptional changes associated with AD, frontotemporal dementia, and related conditions. In parallel, mass spectrometry‐based proteomics will be employed to analyze the composition and structure of protein aggregates found in disease‐specific lesions. These approaches aim to uncover novel biomarkers, delineate disease subtypes, and reduce diagnostic inequities in global dementia research.

### 1.16 Virtual reality (VR) and applied neuroeducation

VR and 3D visualization are being integrated into neuroscience education and public outreach to enhance engagement and comprehension. These immersive tools enable anatomically accurate representations of brain structures and neuropathological lesions using real clinical and research data. In educational settings, they offer interactive training experiences for students and professionals, supporting the development of neuropathological analysis skills. In parallel, their implementation in traveling exhibitions provides an accessible and compelling medium for public science communication, fostering broader awareness and understanding of brain health, function, and disease progression among diverse audiences (Figure 3).

### 1.17 Analysis

The BND, based at the Universidad Politécnica de Pachuca, and the BNC‐UNPHU in the Dominican Republic represent groundbreaking initiatives in Latin America.^5^, ^53^ These centers have successfully established protocols for the acquisition, preservation, and analysis of *post mortem* human brain tissue in accordance with international standards, with a primary focus on AD. Both centers operate as diagnostic and molecular neuropathology research units while also serving as academic training hubs and platforms for public education.

Through a holistic model that integrates science, education, and public outreach, including traveling exhibitions such as “A New Life for the Brain in Mexico” and “The Brain and Neurodegenerative Diseases in the Dominican Republic,” these brain banks are advancing a socially engaged vision of neuroscience. They have also played a key role in increasing the representation of mestizo, Afro‐Caribbean, and Indigenous populations, groups historically excluded from global neuroscience research. This inclusion enriches the genetic and epigenetic diversity of studies on AD and other neurodegenerative conditions, while also challenging the longstanding Eurocentric bias in neuropathological models.

Both brain banks are pioneering the use of emerging technologies, such as AI, in conjunction with the histological techniques developed in‐house. These innovations aim to promote a culture of brain tissue donation in support of accurate diagnosis and molecular research. Furthermore, these institutions serve as critical training grounds for students, technicians, and professionals in neuroscience and molecular pathology (Figure 2) and have become active nodes in international scientific collaboration and science diplomacy.

## 2 CONCLUSIONS

The BND in Mexico and the BNC‐UNPHU at the Universidad Nacional Pedro Henríquez Ureña in the Dominican Republic represent emerging pillars of neuropathological research in Latin America. The development of these neurobanks has enabled the implementation of integrative models that combine brain tissue collection, ethical governance, histopathological diagnostics, scientific education, and social engagement. By focusing particularly on neurodegenerative diseases such as AD, these initiatives make a significant contribution to understanding the biological underpinnings of dementia from a regional, diverse, and historically underrepresented perspective.

The integration of technological innovation, community participation, and ethical regulation positions these neurobanks as catalysts of a new Latin American neuroscience, one that is more autonomous, inclusive, and socially responsive. Their continued consolidation will not only strengthen translational medicine across the region but also help reduce global disparities in biomedical research. Investing in their sustainability and growth ultimately represents a commitment to memory, sovereignty, and a collective legacy for a more equitable understanding of the human brain.

## CONFLICTS OF INTEREST STATEMENT

The authors declare no conflicts of interest. Author disclosures are available in the Supporting Information.

## CONSENT STATEMENT

Consent was unnecessary.

## Supporting information



Supporting Information

## References

[1] Danner B , Gonzalez AD , Corbett WC , et al. Brain banking in the United States and Europe: importance, challenges, and future trends. J Neuropathol Exp Neurol. 2024;83(4):219‐229. doi:10.1093/jnen/nlae014 38506125 PMC10951968

[2] Sosa AL , Brucki SM , Crivelli L , et al. Advancements in dementia research, diagnostics, and care in Latin America: highlights from the 2023 Alzheimer's Association International conference satellite symposium in Mexico City. Alzheimers Dement. 2024;20(7):5009‐5026. doi:10.1002/alz.13850 38801124 PMC11247679

[3] Brownell M , Sehar U , Mukherjee U , Reddy PH . Creating cultural and lifestyle awareness about dementia and co‐morbidities. J Alzheimers Dis Rep. 2024;8(1):747‐764. doi:10.3233/ADR-240043 38746643 PMC11091762

[4] McGlinchey E , Duran‐Aniotz C , Akinyemi R , et al. Biomarkers of neurodegeneration across the Global South. Lancet Healthy Longev. 2024;5(10):100616. doi:10.1016/S2666-7568(24)00132-6 39369726 PMC11540104

[5] Reyes‐Pablo AE , Campa‐Cordoba BB , Luna‐Viramontes NI , et al. National dementia BioBank: a strategy for the diagnosis and study of neurodegenerative diseases in Mexico. J Alzheimers Dis. 2020;76(3):853‐862. doi:10.3233/JAD-191015 32568191

[6] Luna‐Muñoz J . Banco Nacional de Cerebros UNPHU. Una realidad en República Dominicana y el Caribe. Aula Revista de Humanidades y Ciencias Sociales. 2023;69(2):61‐70. doi:10.33413/aulahcs.2023.69i2.259

[7] Dufour BD , Albores‐Gallo L , Luna‐Munoz J , et al. Hispano‐American Brain Bank on neurodevelopmental disorders: an initiative to promote brain banking, research, education, and outreach in the field of neurodevelopmental disorders. Brain Pathol. 2022;32(2):e13019. doi:10.1111/bpa.13019 34515386 PMC8877728

[8] Shepherd CE , Alvendia H , Halliday GM . Brain banking for research into neurodegenerative disorders and ageing. Neurosci Bull. 2019;35(2):283‐288. doi:10.1007/s12264-018-0326-3 30604281 PMC6426907

[9] De Oliveira TC , Secolin R , Lopes‐Cendes I . A review of ancestrality and admixture in Latin America and the Caribbean focusing on native American and African descendant populations. Front Genet. 2023;14:1091269. doi:10.3389/fgene.2023.1091269 36741309 PMC9893294

[10] Llibre‐Guerra JJ , Jiang M , Acosta I , et al. Social determinants of health but not global genetic ancestry predict dementia prevalence in Latin America. Alzheimers Dement. 2024;20(7):4828‐4840. doi:10.1002/alz.14041 38837526 PMC11247688

[11] Yang Z , Wang C , Posadas‐Garcia YS , et al. Fine‐mapping in admixed populations using CARMA‐X, with applications to Latin American studies. Am J Hum Genet. 2025;112(5):1215‐1232. doi:10.1016/j.ajhg.2025.02.020 40147449 PMC12120188

[12] Gonzalez Burchard E , Borrell LN , Choudhry S , et al. Latino populations: a unique opportunity for the study of race, genetics, and social environment in epidemiological research. Am J Public Health. 2005;95(12):2161‐2168. doi:10.2105/AJPH.2005.068668 16257940 PMC1449501

[13] Acosta‐Uribe J , Pina Escudero SD , Cochran JN , et al. Genetic contributions to Alzheimer's disease and frontotemporal dementia in admixed Latin American populations. medRxiv. 2024;2024. doi:10.1101/2024.10.29.24315197 PMC12827047 41586258

[14] Ramos C , Aguillon D , Cordano C , Lopera F . Genetics of dementia: insights from Latin America. Dement Neuropsychol. 2020;14(3):223‐236. doi:10.1590/1980-57642020dn14-030004 32973976 PMC7500810

[15] Caviedes A , Orellana P , Avila‐Rincon C , et al. Epigenetics of dementia remains unraveled in Latin American and Caribbean populations: a call for collaborative efforts. Alzheimers Dement. 2024;20(12):9076‐9078. doi:10.1002/alz.14295 39535483 PMC11667502

[16] Gonzalez‐Gomez R , Legaz A , Moguilner S , et al. Educational disparities in brain health and dementia across Latin America and the United States. Alzheimers Dement. 2024;20(9):5912‐5925. doi:10.1002/alz.14085 39136296 PMC11497666

[17] Acosta‐Uribe J , Aguillon D , Cochran JN , et al. A neurodegenerative disease landscape of rare mutations in Colombia due to founder effects. Genome Med. 2022;14(1):27. doi:10.1186/s13073-022-01035-9 35260199 PMC8902761

[18] Marino‐Ramirez L , Sharma S , Hamilton JM , et al. The Consortium for Genomic Diversity, Ancestry, and Health in Colombia (CODIGO): building local capacity in genomics and bioinformatics. Commun Biol. 2025;8(1):1062. doi:10.1038/s42003-025-08496-9 40676265 PMC12271396

[19] Lopera F , Marino C , Chandrahas AS , et al. Resilience to autosomal dominant Alzheimer's disease in a Reelin‐COLBOS heterozygous man. Nat Med. 2023;29(5):1243‐1252. doi:10.1038/s41591-023-02318-3 37188781 PMC10202812

[20] Tejada Moreno JA , Villegas Lanau A , Madrigal Zapata L , et al. Mutations in SORL1 and MTHFDL1 possibly contribute to the development of Alzheimer's disease in a multigenerational Colombian Family. PLoS One. 2022;17(7):e0269955. doi:10.1371/journal.pone.0269955 35905044 PMC9337667

[21] Frenkel B , Gerasimov E , Gustafson A , et al. Postmortem tissue donation: giving families the ability to choose. J Clin Oncol. 2023;41(3):447‐451. doi:10.1200/JCO.21.02839 36027484

[22] Griffin CP , Carlson MA , Walker MM , Lynam J , Paul CL . ‘I think both of us drew strength from it’: qualitative reflections from next of kin following the death and post‐mortem brain donation of a loved one with brain cancer. Palliat Care Soc Pract. 2024;18:26323524241272106. doi:10.1177/26323524241272106 39165564 PMC11334123

[23] Bilbrey AC , Humber MB , Plowey ED , et al. The impact of Latino values and cultural beliefs on brain donation: results of a pilot study to develop culturally appropriate materials and methods to increase rates of brain donation in this under‐studied patient group. Clin Gerontol. 2018;41(3):237‐248. doi:10.1080/07317115.2017.1373178 29227743 PMC5962259

[24] Namazi H , Mirikermanshahi S . The medicalization of ethics or ethicalization of neuroscience: toward a conceptual re‐examination. IBRO Neurosci Rep. 2024;16:567‐570. doi:10.1016/j.ibneur.2024.04.004 38764540 PMC11101861

[25] Farah MJ . Neuroethics: the ethical, legal, and societal impact of neuroscience. Annu Rev Psychol. 2012;63:571‐591. doi:10.1146/annurev.psych.093008.100438 19575613

[26] Pugh J . Autonomy, rationality, and contemporary bioethics. 1st ed. Oxford University Press; 2020:x. Oxford philosophical monographs. 287 pages. 32396289

[27] Morlett Paredes A , Guarena LA , Stickel AM , Schairer CE , Gonzalez HM . To donate, or not to donate, that is the question: latino insights into brain donation. Alzheimers Dement. 2023;19(4):1274‐1280. doi:10.1002/alz.12755 36029516 PMC9968816

[28] Muramoto O . Informed consent for the diagnosis of brain death: a conceptual argument. Philos Ethics Humanit Med. 2016;11(1):8. doi:10.1186/s13010-016-0042-4 27737717 PMC5062821

[29] Aguila E , Weidmer BA , Illingworth AR , Martinez H . Culturally competent informed‐consent process to evaluate a social policy for older persons with low literacy: the Mexican Case. Sage Open. 2016;6(3). doi:10.1177/2158244016665886 PMC5562373 28824826

[30] Marshall PA . Informed consent in international health research. J Empir Res Hum Res Ethics. 2006;1(1):25‐42. doi:10.1525/jer.2006.1.1.25 19385865

[31] Fields LM , Calvert JD . Informed consent procedures with cognitively impaired patients: a review of ethics and best practices. Psychiatry Clin Neurosci. 2015;69(8):462‐471. doi:10.1111/pcn.12289 25756740

[32] Siminoff LA , Wilson‐Genderson M , Mosavel M , Barker L , Trgina J , Traino HM . Confidentiality in Biobanking research: a comparison of donor and nondonor families' understanding of risks. Genet Test Mol Biomarkers. 2017;21(3):171‐177. doi:10.1089/gtmb.2016.0407 28121471 PMC5367914

[33] Huitinga I , de Goeij M , Klioueva N . Legal and ethical issues in brain banking. Neurosci Bull. 2019;35(2):267‐269. doi:10.1007/s12264-018-0305-8 30390244 PMC6426895

[34] Cash RA , Gutnick R . Casebook on ethical issues in international health research. World Health Organization; 2009:1. https://hdl.loc.gov/loc.gdc/gdcebookspublic.2021763144. electronic resource (209 pages).

[35] Singh A , Arulogun O , Akinyemi J , et al. Biological sample donation and informed consent for neurobiobanking: evidence from a community survey in Ghana and Nigeria. PLoS One. 2022;17(8):e0267705. doi:10.1371/journal.pone.0267705 35951660 PMC9371301

[36] Griffin CP , Bowen JR , Walker MM , Lynam J , Paul CL . Understanding the value of brain donation for research to donors, next‐of‐kin and clinicians: a systematic review. PLoS One. 2023;18(12):e0295438. doi:10.1371/journal.pone.0295438 38117774 PMC10732432

[37] Sharp C , Randhawa G . Altruism, gift giving and reciprocity in organ donation: a review of cultural perspectives and challenges of the concepts. Transplant Rev (Orlando). 2014;28(4):163‐168. doi:10.1016/j.trre.2014.05.001 24973193

[38] Rush A , Weil C , Siminoff L , et al. The experts speak: challenges in banking brain tissue for research. Biopreserv Biobank. 2024;22(2):179‐184. doi:10.1089/bio.2024.29135.ajr 38621226 PMC11265615

[39] Shankar SK , Mahadevan A . Brain banking in India: relevance in current day practice. Indian J Pathol Microbiol. 2022;65(Supplement):S218‐S225. doi:10.4103/ijpm.ijpm_113_22 35562152

[40] Cattaneo C , Urakcheeva I , Giacomini G , et al. Attitude and concerns of healthy individuals regarding post‐mortem brain donation. A qualitative study on a nation‐wide sample in Italy. BMC Med Ethics. 2023;24(1):104. doi:10.1186/s12910-023-00980-3 38012766 PMC10683267

[41] Lambe S , Cantwell N , Islam F , Horvath K , Jefferson AL . Perceptions, knowledge, incentives, and barriers of brain donation among African American elders enrolled in an Alzheimer's research program. Gerontologist. 2011;51(1):28‐38. doi:10.1093/geront/gnq063 20679141 PMC3106364

[42] Lin MP , Jowsey T , Curtis MA . Why people donate their brain to science: a systematic review. Cell Tissue Bank. 2019;20(4):447‐466. doi:10.1007/s10561-019-09786-3 31538265 PMC6863784

[43] Ferreira EKGD , Silveira GF . Classification and counting of cells in brightfield microscopy images: an application of convolutional neural networks. Scientific Reports. 2024;14(1):9031. doi:10.1038/s41598-024-59625-z 38641688 PMC11031575

[44] Moloney CM , Lowe VJ , Murray ME . Visualization of neurofibrillary tangle maturity in Alzheimer's disease: a clinicopathologic perspective for biomarker research. Alzheimers Dement. 2021;17(9):1554‐1574. doi:10.1002/alz.12321 33797838 PMC8478697

[45] Switzer RC, 3rd . Application of silver degeneration stains for neurotoxicity testing. Toxicol Pathol. 2000;28(1):70‐83. doi:10.1177/019262330002800109 10668992

[46] King A , Bodi I , Troakes C . The neuropathological diagnosis of alzheimer's disease‐the challenges of pathological mimics and concomitant pathology. Brain Sci. 2020;10(8):479. doi:10.3390/brainsci10080479 32722332 PMC7463915

[47] Braak H , Braak E . [Morphological changes in the human cerebral cortex in dementia]. J Hirnforsch. 1991;32(3):277‐282. Morphologische Veranderungen der menschlichen Endhirnrinde bei Demenz. 1779131

[48] Braak H , Braak E . Neuropathological stageing of Alzheimer‐related changes. Acta Neuropathol. 1991;82(4):239‐259. doi:10.1007/bf00308809 1759558

[49] Luna‐Munoz J , Chavez‐Macias L , Garcia‐Sierra F , Mena R . Earliest stages of tau conformational changes are related to the appearance of a sequence of specific phospho‐dependent tau epitopes in Alzheimer's disease. J Alzheimers Dis. 2007;12(4):365‐375. doi:10.3233/jad-2007-12410 18198423

[50] Luna‐Munoz J , Peralta‐Ramirez J , Chavez‐Macias L , Harrington CR , Wischik CM , Mena R . Thiazin red as a neuropathological tool for the rapid diagnosis of Alzheimer's disease in tissue imprints. Acta Neuropathol. 2008;116(5):507‐515. doi:10.1007/s00401-008-0431-x 18810470

[51] Luna‐Viramontes NI , Campa‐Cordoba BB , Ontiveros‐Torres MA , et al. PHF‐Core Tau as the potential initiating event for tau pathology in Alzheimer's disease. Front Cell Neurosci. 2020;14:247. doi:10.3389/fncel.2020.00247 33132840 PMC7511711

[52] Mukherjee A , Al‐Lahham R , Corkins ME , et al. Identification of multicolor fluorescent probes for heterogeneous abeta deposits in Alzheimer's disease. Front Aging Neurosci. 2021;13:802614. doi:10.3389/fnagi.2021.802614 35185519 PMC8852231

[53] BNC‐UNPHU . Banco Nacional de Cerebros. Universidad Nacional Pedro Henriquez Ureña. https://bnc.unphu.edu.do/

